# A decade-long seamless-continuity daily L-band soil moisture product derived from SMOS observations since 2010

**DOI:** 10.1038/s41597-026-06756-9

**Published:** 2026-02-11

**Authors:** Yu Bai, Li Jia, Tianjie Zhao, Zhiqing Peng, Jingyao Zheng, Chaolei Zheng, Ping Tang, Jiancheng Shi

**Affiliations:** 1 State Key Laboratory of Remote Sensing and Digital Earth, Aerospace Information Research Institute, Chinese Academy of Sciences, Beijing, China; 2 Department of Earth System Science, Institute for Global Change Studies, Tsinghua University, Beijing, China; 3 Aerospace Information Research Institute, Chinese Academy of Sciences, Beijing, China; 4 National Space Science Center, Chinese Academy of Sciences, Beijing, China

**Keywords:** Environmental impact, Hydrology

## Abstract

Soil moisture (SM) is a fundamental state variable in the Earth system that governs water, energy, and carbon exchanges between land and atmosphere. Current satellite-derived SM products, such as SMOS (Soil Moisture and Ocean Salinity) SM product, exhibit pronounced spatial and temporal discontinuities caused by orbital coverage, radio frequency interference, and retrieval failures, which severely limit their utility to long-term eco-hydrological studies. This study introduces a fully automated gap-filling method that combines Discrete Cosine Transformation with Partial Least Squares (DCT-PLS). The method exploits the intrinsic spatiotemporal coherence of the original SMOS Multi-Temporal and Multi-Angular (MTMA) product without relying on any external ancillary data. It was applied to the entire SMOS MTMA archive to generate a seamless-continuity product (MTMA-SC_SM) at 25 km and daily resolution. Reconstruction of the synthetic gaps confirmed its fidelity (correlation coefficient, R > 0.9; root mean squared error, RMSE, and mean absolute error, MAE < 0.04 m³/m³). When evaluated against the 22 *in-situ* SM networks, the MTMA-SC_SM product achieved an overall R > 0.7 and unbiased RMSE = 0.057 m³/m³, performing comparably to the original SMOS MTMA retrievals. Spatially, the seamless product preserved mesoscale patterns and seasonal amplitudes across all climate zones, with no discernible boundary artifacts around reconstructed regions. The MTMA-SC_SM dataset constitutes the first decade-long, gap-free, global daily L-band SM record, providing a robust foundation for climate trend assessment and land surface modelling at the global scale.

## Background & Summary

Soil moisture is a key variable in the global hydrological cycle system and plays a crucial role in research on global energy cycling and environmental change^1–6^. Microwave remote sensing has become an important means of monitoring global surface soil moisture due to its advantages of being all-day, all-weather and sensitive to the nuances of soil moisture changes^1,7–9^. This technology has been embraced for its ability to penetrate vegetation and detect even subtle changes in soil moisture, with the L-band in particular being esteemed for its monitoring capabilities^10–12^.

At present, the satellite-derived global daily soil moisture products mainly come from active and passive satellite sensors, such as ASCAT (Advanced Scatterometer), AMSR-E/2 (Advanced Microwave Scanning Radiometer-EOS/2)^13,14^, FengYun-3 MWRI (Microwave Radiation Imager)^15^, SMOS (Soil Moisture and Ocean Salinity)^16,17^, and SMAP (Soil Moisture Active and Passive)^18–21^. However, due to satellite orbit coverage issues, potential satellite malfunction, radio frequency interference (RFI), and challenges associated with retrieval algorithms (the error accumulation in the soil moisture retrievals during the decoupling of vegetation and soil information via retrieval algorithms, or the non-convergence of the solution results caused by the nonlinear retrieval algorithms), there are gaps in these daily global soil moisture products. These data voids can limit the application of soil moisture products for modelling global climate change and land surface processes^22–26^, especially for daily temporal analyses. For example, global (or large-scale) evapotranspiration estimation or spatial boundary capture of saturated-unsaturated zone for hydrological models (such as SWAT model) require spatiotemporal continuous soil moisture data as input. However, the presence of gaps may affect the simulation process. Gaps can lead to the fragmentation of drought range estimated based on soil moisture data, making it difficult to accurately determine the continuous distribution of drought, which in turn affects the estimation of drought areas. In addition, assessing the expansion or reversal trend of desertified areas also requires spatiotemporal continuous soil moisture data, and gaps in soil moisture data can lead to ‘breakpoints’ in desertification trends, resulting in misjudgments.

To tackle the issue of data gaps in satellite-derived daily soil moisture products, researchers have undertaken extensive investigations into the reconstruction of missing data. Several types of reconstruction methods have been explored, each with its unique focus and application. (1) One such category is the spatial-based reconstruction methods^27,28^. These methods encompass a range of techniques including the nearest neighbor interpolation, inverse distance weighting interpolation^29^, the Tyson polygon method^30,31^, spline method, and the kriging method^32^. These techniques are particularly adept at utilizing the spatial correlation present within the data, employing the relationships between nearby valid measurements to infer values for missing data points. However, these spatial methods inherently prioritize spatial relationships over temporal dynamics. This focus, although beneficial for highlighting spatial patterns, may result in a less pronounced representation of temporal variations. In scenarios where the landscape is highly variable or where there is a significant absence of spatial data, the performance of these methods might not fully meet the expectations set by more homogenous conditions. (2) The temporal methods capitalize on the intrinsic dynamics of time series data to perform reconstructions^33–35^, including a variety of techniques such as the linear interpolation^36^, spline interpolation^37^, the time series harmonic analysis through Fourier transform^38^, and singular spectrum analysis in an iterative reconstruction framework^39^. These temporal methods stand out for their ability to capture the evolving patterns within time series data. However, the efficacy of these methods is particularly sensitive to the selection of the time interval for analysis. When intervals are too short, the method may not adequately capture the necessary temporal correlations, potentially leading to less effective reconstructions. Conversely, when the time interval is overly long, the surface parameters can undergo substantial variations, thereby compromising the reliability of reconstruction results^33^. (3) Spatiotemporal methods, relying on the synthesis of spatial and temporal dimensions, integrate one-dimensional temporal dynamics with two-dimensional spatial patterns to offer a multifaceted approach to addressing data gaps^40,41^. Although Borak *et al*.^42^ and Pede *et al*.^43^ have confirmed the superior capability of spatiotemporal information in reconstructing missing data compared to single-dimension (temporal or spatial) approaches, it is essential to acknowledge that the incorporation of auxiliary data in these spatiotemporal-based approaches introduces uncertainties that can impact the reconstruction results^44^. (4) Machine learning has introduced a transformative approach to soil moisture data reconstruction, with methodologies such as neural networks, random forest, deep learning network and decision trees being utilized to create long-term, continuous datasets from AMSR-E/2^24,45,46^, SMAP^47^, and FengYun-3^48^ sensors. While these models excel at capturing complex patterns, their performance is inherently linked to the quality of the training data and the associated feature parameters. As a result, the inherent variability in these parameters must be carefully considered to ensure the robustness of such models^49^.

Overall, the current research predominantly focuses on the reconstruction of global seamless daily soil moisture products at high frequencies (such as the C/X bands for AMSR-E/2 sensors)^24,45,46^, while limited attention is given to the reconstruction of soil moisture products at lower frequencies (L-band). Compared to high-frequency (C/X-band) microwaves, low-frequency (L-band) microwaves can penetrate sparse and moderately dense vegetation to obtain soil information at a depth of 0~5 cm^9,50^. The L-band is considered the best band for obtaining surface soil moisture due to its higher sensitivity to surface soil moisture^11,50^. Zheng *et al*. found that the overall behavior of L-band soil moisture products was better than that of high-frequency (the C/X bands for AMSR-E/2 and FengYun-3 sensors) soil moisture products^11^. As the vegetation becomes denser, the performance of the low-frequency (L-band) soil moisture product is superior to that of the high-frequency products^50^. Moreover, to mitigate the influence of external auxiliary information and effectively exploit the inherent spatiotemporal characteristics of the dataset, the Discrete Cosine Transformation-Partial Least Squares (DCT-PLS) method^51^ is used to reconstruct missing data in this study. The DCT-PLS method belongs to a spatiotemporal method (as described in category 3 of traditional reconstruction methods mentioned above). However, compared to current spatiotemporal methods, the DCT-PLS method is independent of auxiliary data and relies on the spatiotemporal characteristics of the dataset itself as a basis to fill gaps^51^. In this study, we applied the DCT-PLS method to reconstruct missing data in soil moisture products generated by the world’s first SMOS L-band sensor, resulting in a comprehensive, global spatiotemporal seamless daily soil moisture product (2010 to 2020) at L-band.

## Methods

### The DCT-PLS method

Maximizing the utilization of temporal continuity and spatial correlation inherent in the dataset itself, while minimizing errors introduced by auxiliary information, is a crucial strategy for achieving seamless spatio-temporal filling. One feasible approach for this is the utilization of the DCT-PLS method^51^, which leverages the one-dimensional time series continuity and two-dimensional spatial consistency information of the original soil moisture product to reconstruct missing data regions. The objective of this study is to employ the DCT-PLS method to fill the gaps of SMOS L-band soil moisture products, and then to obtain long-term global spatiotemporal seamless daily soil moisture products, thereby enhancing the usability of this soil moisture product for spatio-temporal characteristics analysis and the assessment of global climate change in specific regions.

The DCT-PLS method mainly uses the partial least square method to reconstruct missing data:

1 $$F(\hat{y})={RSS}+s\cdot P(\hat{y})={{\rm{||}}\hat{y}-y{\rm{||}}}^{2}+s\cdot P(\hat{y})$$

where, ||·|| represents the Euclid norm, RSS represents the residual sum of squares and is used to measure the fidelity of the smoothing results, $\hat{y}$ represents the smoothed soil moisture data, $P\left(\hat{y}\right)$ represents the roughness (smoothness) of the data without smoothing, $s$ represents the positive real number used to control the smoothness and the smoothness degree of $\hat{y}$ increases with the increase of the parameter $s$. The primary objective of the DCT-PLS method is to achieve the smoothing of the original data $y$ by determining the optimal solution of $\hat{y}$.

Based on second-order difference, $P\left(\hat{y}\right)$ can be expressed by:

2 $$P\left(\hat{y}\right)={{\rm{||}}D\cdot \hat{y}{\rm{||}}}^{2}$$

3 $$D=\left(\begin{array}{crccccc}-1 & 1 &  &  &  &  & \\ 1 & -2 & 1 &  &  &  & \\  & 1 & -2 &  &  &  & \\  &  &  & \ddots  &  &  & \\  &  &  &  & -2 & 1 & \\  &  &  &  & 1 & -2 & 1\\  &  &  &  &  & 1 & -1\end{array}\right)$$

where, $D$ represents the tridiagonal matrix. Given that the pixel distances in the soil moisture products generated using the MTMA method are equal, $D$ can be represented by Eq. (3). By substituting Eq. (2) into Eq. (1), the linear relationship between the original soil moisture data and the smoothed soil moisture data can be obtained:

4 $$y=\left({I}_{n}+s\cdot {D}^{T}\cdot D\right)\cdot \hat{y}$$

5 $$D=U\cdot \varLambda \cdot {U}^{-1}$$

where, ${I}_{n}$ represents the diagonal matrix of $n\times n$, ${D}^{T}$ is the transposition of the matrix $D$, $U$ is the unitary matrix and ${U}^{-1}={U}^{T}$, $U\cdot {U}^{T}={I}_{n}$, $\varLambda$ ($\varLambda ={diag}\left({\lambda }_{1},{\lambda }_{2},\ldots ,{\lambda }_{n}\right)$, ${\lambda }_{i}=-2+2\cdot \cos \,(\left(i-1\right)\cdot \pi /n)$) is the diagonal matrix containing the eigenvalue of matrix $D$. Equation (5) is obtained by eigen-decomposition of matrix $D$.

Equation (4) can be rewritten as:

6 $$\hat{y}=U\cdot {\left({I}_{n}+s\cdot {\varLambda }^{2}\right)}^{-1}\cdot {U}^{T}\cdot y=U\cdot \varGamma \cdot {U}^{T}\cdot y$$

where, $U$ and ${U}^{T}$ are the discrete cosine transform (DCT) matrix and the inverse matrix of the discrete cosine transform (IDCT) matrix of $n\times n$ respectively, Γ is the diagonal matrix containing the parameter $s$. When smoothing one-dimensional time series data, ${\varGamma }_{i}={\left[1+s\cdot {\left(2-2\cdot \cos (\left(i-1\right)\cdot \pi /n)\right)}^{2}\right]}^{-1}$, and when smoothing three-dimensional spatiotemporal data, ${\varGamma }_{{i}_{1}{,i}_{2},{i}_{3}}={[1+s\cdot (\mathop{\sum }\limits_{j=1}^{3}{\left(2-\cos (\left({i}_{j}-1\right)\cdot \pi /{n}_{j})\right)}^{2})]}^{-1}$. Equation (6) also can be rewritten as:

7 $$\hat{y}={IDCT}\left(\varGamma \cdot {DCT}\left(y\right)\right)$$

According to Eq. (7), the reconstruction process of missing soil moisture using the DCT-PLS method involves performing DCT and IDCT transformations sequentially on the original soil moisture data. The performance of reconstruction results is primarily influenced by the parameter $s$. The parameter $s$ is typically determined using the generalized cross-validation method, which involves iterative optimization to minimize the smoothing error^51^.

### Evaluation by generating artificial gaps

This study assesses the performance of the DCT-PLS method in spatiotemporal reconstruction of soil moisture through simulation experiments with artificially generated gaps.

1. time-series validation
  The performance of the DCT-PLS method in reconstructing simulated missing time-series soil moisture is assessed based on *in-situ* data from 22 ground soil moisture observation networks in this study. For each soil moisture network, one-third of the *in-situ* data are randomly selected and replaced with ‘NaN’ values (i.e., as gaps). The DCT-PLS method is then used to reconstruct the soil moisture values at these locations. It should be noted that one-third of the randomly selected *in-situ* data from each soil moisture network were used as the ‘true value’ to assess the reconstruction results.
2. Simulated missing-region validation

In addition to assessing the time series consistency between the soil moisture reconstructed by the DCT-PLS method and the *in-situ* data, it is also necessary to assess the DCT-PLS method’s capability in reconstructing spatially continuous soil moisture. To further demonstrate this, the study evaluated the performance of the DCT-PLS method in reconstructing soil moisture over regions with missing data.

**Fig. 1:**
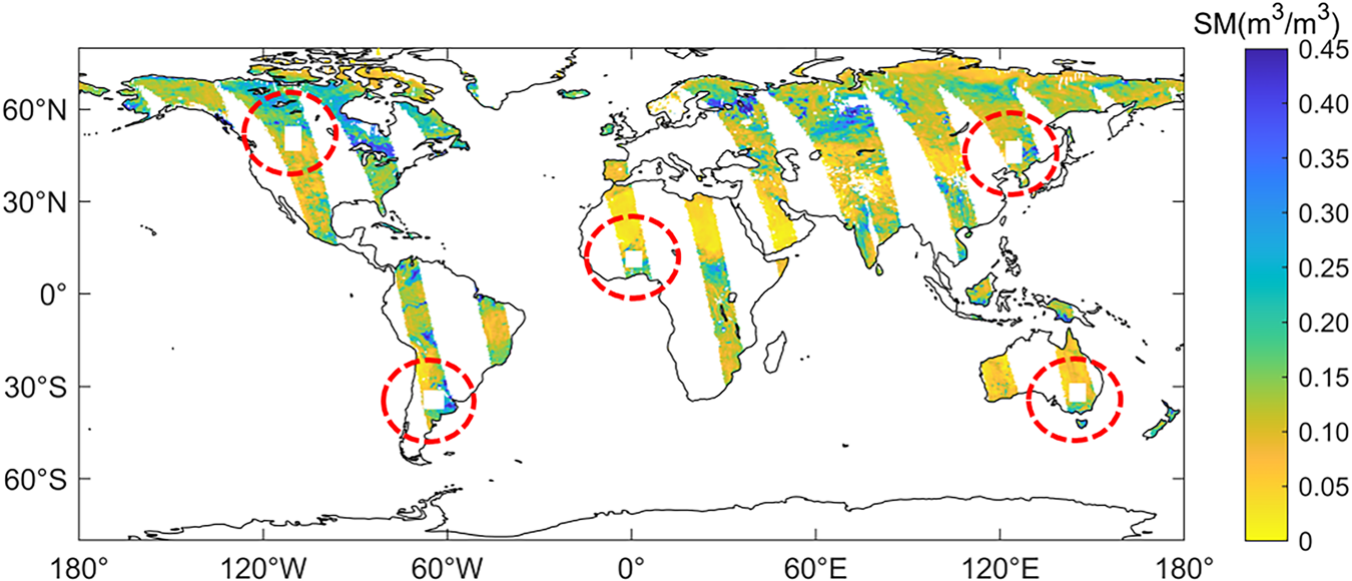
Global spatial distribution of original soil moisture with five simulated missing data areas in 1 June 2017.

Figure 1 shows an example of the five simulated missing data regions selected based on the original SMOS MTMA daily soil moisture product. The values within these regions are replaced with ‘NaN’. The soil moisture values in these areas were then reconstructed using the DCT-PLS method. This study compares the reconstructed values with the original values to evaluate the fidelity of the DCT-PLS method in reconstructing spatial continuity information.

### Accuracy metrics

*In-situ* validation is the most reliable method for evaluating the applicability of the DCT-PLS method^52–54^. To quantitatively evaluate the performance of the SMOS MTMA-SC_SM products obtained using the DCT-PLS method, five validation indices are used to compare *in-situ* soil moisture and reconstructed soil moisture in this study. These indices include the mean absolute error (MAE), the unbiased root mean squared error (ubRMSE), root mean squared error (RMSE), the Bias, and the correlation coefficient (R). The equations for calculating these five indices are as follows:

8 $${MAE}=\frac{\mathop{\sum }\limits_{i=1}^{n}|{{SM}}_{i}^{{est}}-{{SM}}_{i}^{{obs}}|}{n}$$

9 $$R=\frac{\mathop{\sum }\limits_{i=1}^{n}\left({{SM}}_{i}^{{est}}-E\left[{{SM}}^{{est}}\right]\right)\cdot \left({{SM}}_{i}^{{obs}}-E\left[{{SM}}^{{obs}}\right]\right)}{\sqrt{{\mathop{\sum }\limits_{i=1}^{n}\left({{SM}}_{i}^{{est}}-E\left[{{SM}}^{{est}}\right]\right)}^{2}\cdot {\mathop{\sum }\limits_{i=1}^{n}\left({{SM}}_{i}^{{obs}}-E\left[{{SM}}^{{obs}}\right]\right)}^{2}}}$$

10 $${RMSE}=\sqrt{E\left[{\left({{SM}}^{{est}}-{{SM}}^{{obs}}\right)}^{2}\right]}$$

11 $${Bias}=E\left[{{SM}}^{{est}}\right]-E\left[{{SM}}^{{obs}}\right]$$

12 $${ubRMSE}=\sqrt{{{RMSE}}^{2}-{{Bias}}^{2}}$$

where, E [.] represents the expectation or linear averaging operator, ${{SM}}^{{est}}$ represents the estimated soil moisture, ${{SM}}^{{obs}}$ represents the *in-situ* soil moisture.

### SMOS MTMA soil moisture products

In this study, soil moisture products (referred to as MTMA_SM) from June 2010 to December 2020 are obtained using the MTMA method^17,55^ based on SMOS H-pol multi-angular brightness temperature data. The MTMA_SM product exhibits a temporal resolution of one day and a spatial resolution of 25 km. Compared to SMOS Level 2 (L2)/Level 3 (L3)^34,56^ and SMOS-IC^16,57^ products, the global daily land coverage of MTMA_SM products is higher. Figure 1 presents the global spatial distribution of MTMA_SM products on 1 June, 2017. However, due to the impact of satellite orbit, satellite malfunction, radio frequency interference (RFI), and ineffective retrievals caused by the uncertainty of the soil moisture retrieval algorithm itself, there is a significant phenomenon of missing data in MTMA_SM daily products on the Earth’s land surface (Fig. 1). Taking 2017 as an example, the average land surface coverage rate of MTMA_SM daily products ranges from 39.42% to 50.92% by statistics.

**Fig. 2:**
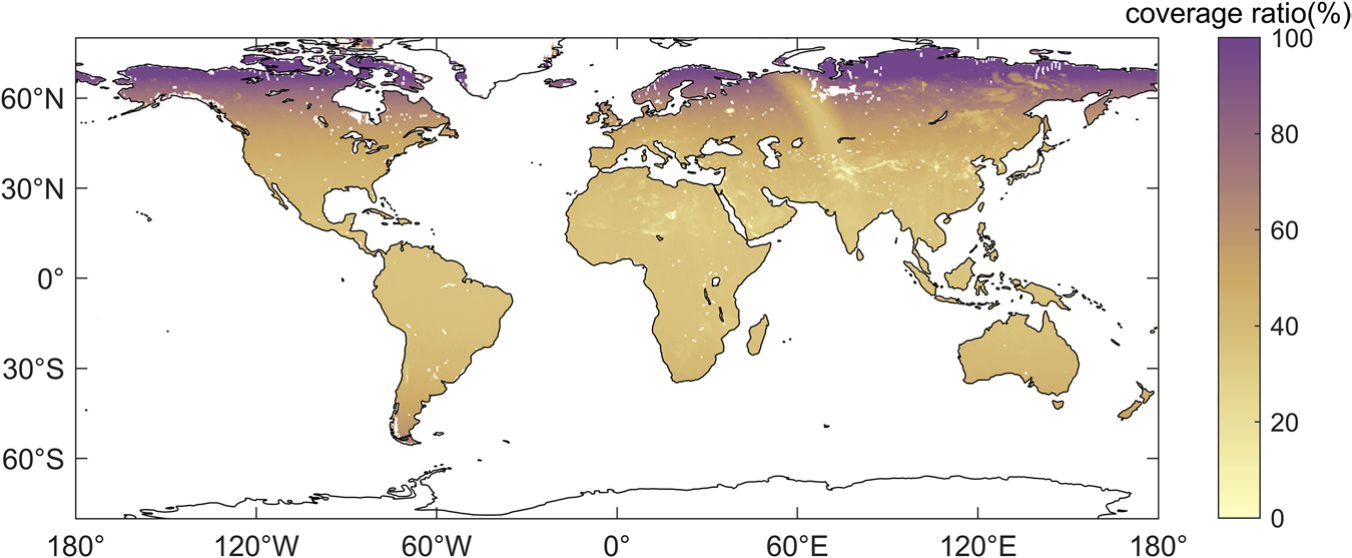
Spatial distribution of coverage rate of soil moisture retrievals in each pixel in 2017.

In addition, Fig. 2 also illustrates the global spatial distribution of coverage rate of soil moisture retrievals for each pixel on the global land surface in 2017. It is evident that the coverage rate of soil moisture retrieval results for each pixel varies significantly with latitude, with the higher coverage rate in high latitude areas due to shorter satellite revisit periods, and lower coverage rate in low latitude areas due to longer satellite revisit periods. Moreover, the global average land surface coverage rate of the MTMA_SM daily products was calculated to be 45.98%. It is clear that the spatiotemporal discontinuity of the SMOS MTMA_SM product makes it unsuitable for subsequent spatiotemporal feature analysis.

### *In-situ* soil moisture measurements

**Fig. 3:**
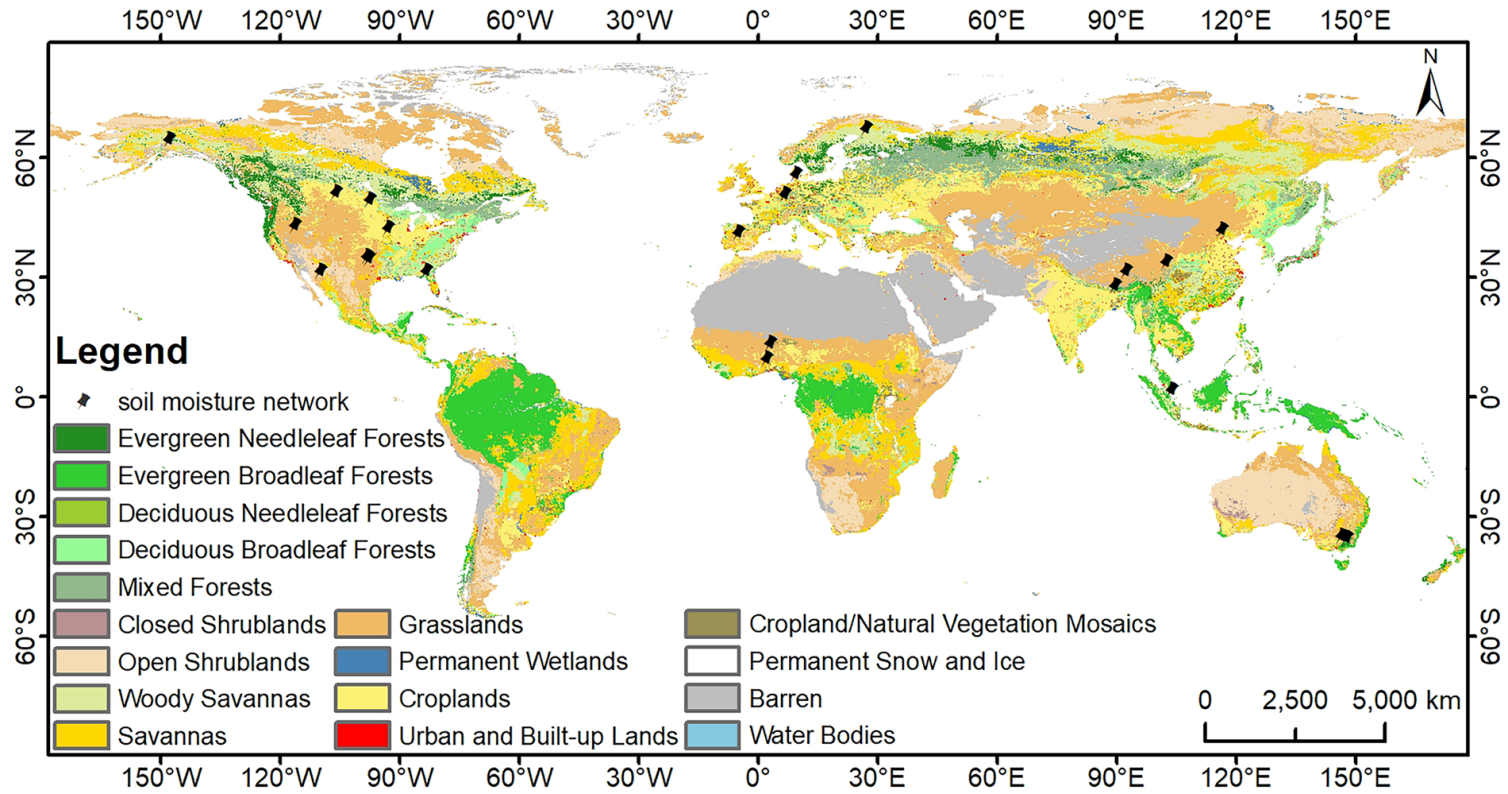
Spatial distribution of the location of the ground soil moisture network.

In this study, *in-situ* soil moisture data are collected from 22 soil moisture observation networks (a total of 398 sites) from the International Soil Moisture Network (ISMN, https://ismn.meaemaearth/en/) and the Long-Term Agroecosystem Research (LTAR) network to quantitatively evaluate the performance of the reconstructed soil moisture. Figure 3 presents the spatial distribution of all ground validation networks used in this study. These soil moisture networks are distributed across five continents (Asia, Europe, Africa, North America, Oceania) in different climatic regions and cover the period from 2010 to 2019. More details of each soil moisture network, including name, land cover type, measuring depth, etc., are given in Table 1. The MTMA_SM products are mainly derived using the SMOS brightness temperature data at 6:00 A.M. Consequently, the corresponding *in-situ* soil moisture data were extracted at the same time to ensure consistency. The *in-situ* measurements are primarily used to evaluate the reliability of the DCT-PLS method and the accuracy of the reconstructed soil moisture.

**Table 1:** The information of *in-situ* soil moisture observation networks used for validation.

Network/Sites	Country	Land Cover	Total sites	Measuring depth	References
AMMA-CATCH (Benin)	Benin	savannas	4	0~5 cm	^60^
AMMA-CATCH (Niger)	Niger	grasslands	3	0~5 cm	^60^
BNZ-LTER	America	woody savannas	10	0~5 cm	^61^
FMI	Finland	woody savannas	22	0~5 cm	^62^
HOBE	Denmark	croplands	32	0~5 cm	^63^
MySMNet	Malaysia	woody savannas and evergreen broadleaf forest	7	0~5 cm	^64^
RISMA (Manitoba)	Canada	croplands and cropland/natural vegetation mosaics	9	0~5 cm	^65^
RISMA (Saskatchewan)	Canada	croplands and cropland/natural vegetation mosaics	4	0~5 cm	^65^
SMN-SDR	China	grasslands	34	0~3 cm	^11,66^
TERENO	Germany	croplands, urban areas, buildings, and mixed forests	4	0~5 cm	^67^
Reynolds Creek	America	grasslands and croplands	18	0~5 cm	^68^
REMEDHUS	Spain	croplands	20	0~5 cm	^69^
Yanco	Australia	grasslands and croplands	12	0~5 cm	^70^
Kyeamba	Australia	croplands	7	0~5 cm	^70^
South Fork	America	croplands	20	0~5 cm	^71^
Fort Cobb	America	grasslands and croplands	14	0~5 cm	^72,73^
Little River	America	croplands and woody savannas	33	0~5 cm	^74,75^
Little Washita	America	grasslands	19	0~5 cm	^72,73^
Walnut Gulch	America	shrub open	29	0~5 cm	^76^
Ali	China	bare area	20	0~5 cm	^77^
Naqu	China	grasslands	57	0~5 cm	^78^
Maqu	China	grasslands	20	0~5 cm	^79^

## Data Records

The SMOS MTMA-SC global daily soil moisture dataset (2010 to 2020)^58^ with a spatial resolution of 25 km (projection type: the EASE-GRID 2.0) is located at 10.11888/Terre.tpdc.303001. All data are stored in NetCDF format, and each file contains variables including soil moisture (SM, m^3^/m^3^), latitude (lat, °), and longitude (lon, °). The file name is “MTMA-SC_SMOS_25KM_YYYYMMDD_V01.nc”, where “MTMA” represents the Multi-Temporal and Multi-Angular method, “SC” represents the seamless-continuity product, “SMOS” represents the Soil Moisture and Ocean Salinity, “YYYY” represents the year, “MM” stands for the month, “DD” stands for the day, “25KM” stands for the spatial resolution of 25 km, and “V01” stands for the version.

## Technical Validation

### Performance assessment of the DCT-PLS method

The following analyses are presented to support the technical reliability of the DCT-PLS method.

#### Experiment with simulated missing data in time series

This section focuses on evaluating the time series reconstruction capability of the DCT-PLS method using *in-situ* soil moisture data from 22 soil moisture observation networks.

**Fig. 4:**
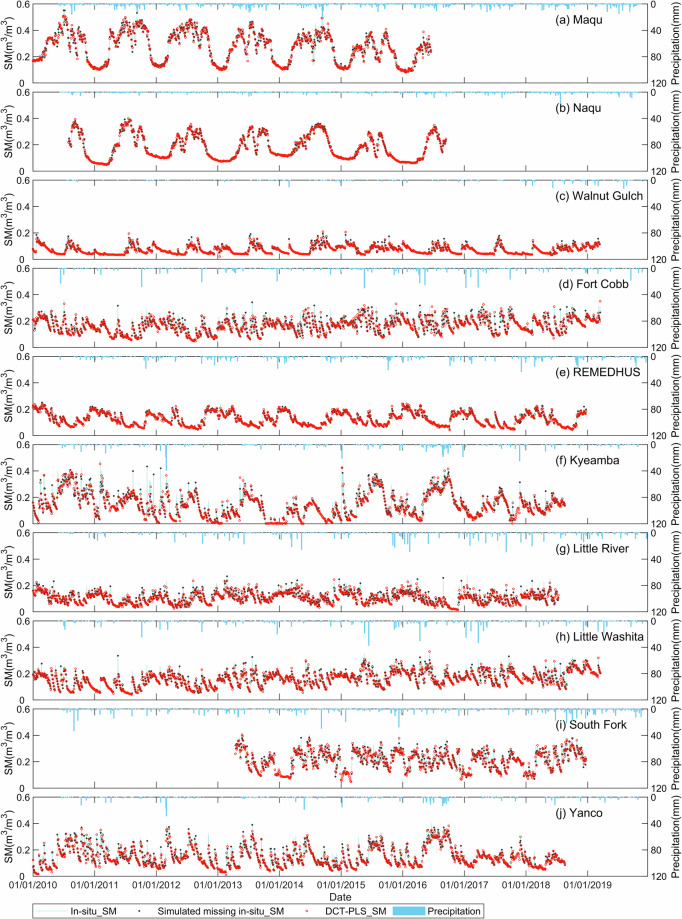
Comparison between the time series of *in-situ* soil moisture and reconstructed soil moisture in 2010 to 2019 at different ground validation networks (the pink hollow diamond represents the *in-situ* soil moisture, and the blue hollow circle represents the reconstructed soil moisture based on the DCT-PLS method).

The time series of *in-situ* and reconstructed soil moisture for 10 soil moisture observation networks (Naqu, Maqu, Yanco, Kyeamba, Little River, Little Washita, South Fork, Fort Cobb, REMEDHUS, and Walnut Gulch) from 1 January 2010 to 31 December 2019 are shown in Fig. 4a–j. The displayed results in Fig. 4a–j correspond to the actual observation periods for each respective ground soil moisture network. In Fig. 4a–j, it was found that both the original and reconstructed time series soil moisture can clearly reveal the interannual change characteristics of soil moisture over most of the soil moisture networks. The reconstructed soil moisture, obtained using the DCT-PLS method, has a good temporal consistency with the *in-situ* soil moisture and effectively captures the dynamic changes in soil moisture. Relatively low soil moisture values occur mainly in winter, while relatively high soil moisture values occur mainly in rainy summer.

The accuracy metrics of the reconstructed soil moisture at 22 ground soil moisture networks are listed in Table 2. In Table 2, it can be found that the correlation between the reconstructed soil moisture and the *in-situ* data exceeds 0.9 at all the soil moisture networks, especially in the Naqu, Maqu, and Reynolds Creeks networks where the R even exceeds 0.99. However, in certain soil moisture networks (such as the Little River and Fort Cobb networks), where soil moisture is significantly influenced by irrigation or rainfall, the consistency between reconstructed and *in-situ* soil moisture is slightly lower. This indicates that the soil moisture reconstructed by the DCT-PLS method may not respond effectively to sudden rainfall or irrigation events. In addition, the RMSE of the reconstructed soil moisture is less than 0.03 m^3^/m^3^ at all soil moisture networks, and the MAE is even less than 0.02 m^3^/m^3^, which is lower than the expected accuracy (0.04 m^3^/m^3^) of the SMOS satellite. According to the accuracy indicators of combining data from all the networks together in Table 2, the overall R, RMSE and MAE of the reconstructed soil moisture are 0.963, 0.015 m^3^/m^3^, and 0.008 m^3^/m^3^ respectively, indicating that the overall consistency between the reconstructed soil moisture using the DCT-PLS method and the measured soil moisture data is very well.

**Table 2:** Quantitative evaluation results of reconstructed soil moisture based on ground soil moisture networks.

Network/Sites	R	RMSE(m^3^/m^3^)	MAE(m^3^/m^3^)	Number
REMEDHUS	0.985	0.009	0.005	1086
Yanco	0.964	0.020	0.010	1030
Kyeamba	0.951	0.029	0.013	1001
South Fork	0.952	0.020	0.012	634
Fort Cobb	0.924	0.021	0.010	1123
Little River	0.911	0.018	0.010	1041
Little Washita	0.944	0.019	0.009	1124
Walnut Gulch	0.967	0.008	0.004	1109
Maqu	0.990	0.016	0.010	786
Naqu	0.996	0.008	0.005	748
Ali	0.979	0.005	0.002	715
Benin	0.960	0.021	0.011	584
Niger	0.921	0.013	0.005	608
BNZ-LTER	0.995	0.006	0.003	366
FMI	0.963	0.010	0.006	852
HOBE	0.976	0.010	0.007	1120
MySMNet	0.930	0.021	0.014	193
RISMA (Manitoba)	0.966	0.019	0.011	438
RISMA (Saskatchewan)	0.960	0.018	0.008	437
SMN-SDR	0.975	0.011	0.006	159
TERENO	0.985	0.013	0.007	924
Reynolds Creek	0.991	0.009	0.005	974
All networks	0.963	0.015	0.008	17052

In general, compared with the time series *in-situ* soil moisture from 2010 to 2019 at all soil moisture networks, the soil moisture reconstructed based on the DCT-PLS method can stably respond to the temporal variation characteristics of soil moisture, which is crucial for the analysis of time series or seasonal changes of soil moisture in specific regions. The aforementioned simulation experiment to reconstruct missing soil moisture data in time series based on *in-situ* data has demonstrated the robustness and reliability of soil moisture reconstruction using the DCT-PLS method.

#### Experiment with simulated missing data regions

**Fig. 5:**
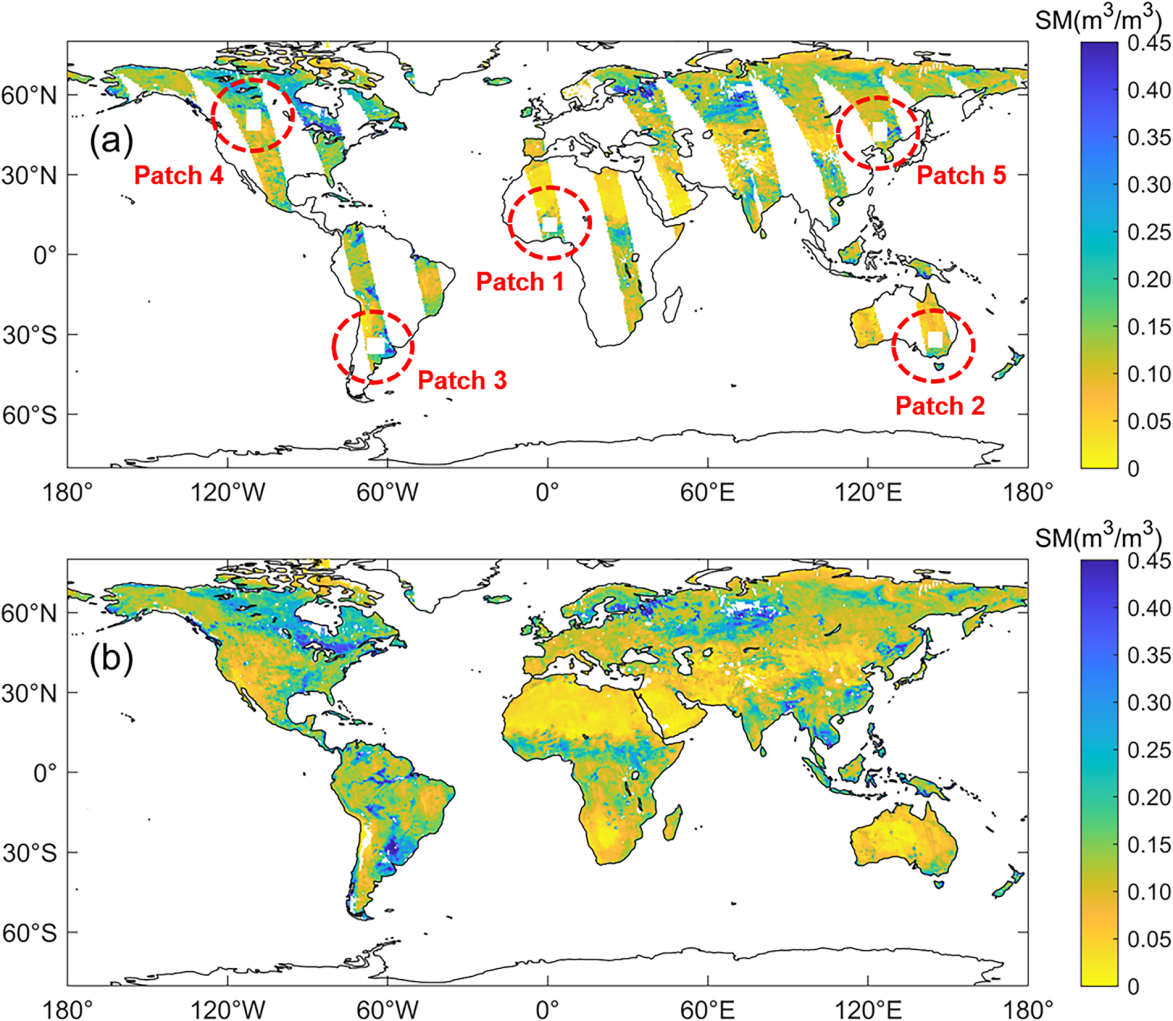
Global spatial distribution of original soil moisture and reconstructed soil moisture with five simulated missing data areas in 1 June 2017: (**a**) Global spatial distribution of original soil moisture with five simulated missing data areas (square area) and (**b**) global spatial distribution of reconstructed soil moisture.

In this study, five simulated missing data regions were selected globally using the original SMOS MTMA soil moisture product based on June 1, 2017 (Fig. 5a). The reliability of the DCT-PLS method in reconstructing spatially continuous soil moisture capability was then evaluated by comparing the reconstructed soil moisture with the original MTMA_SM data.

**Fig. 6:**
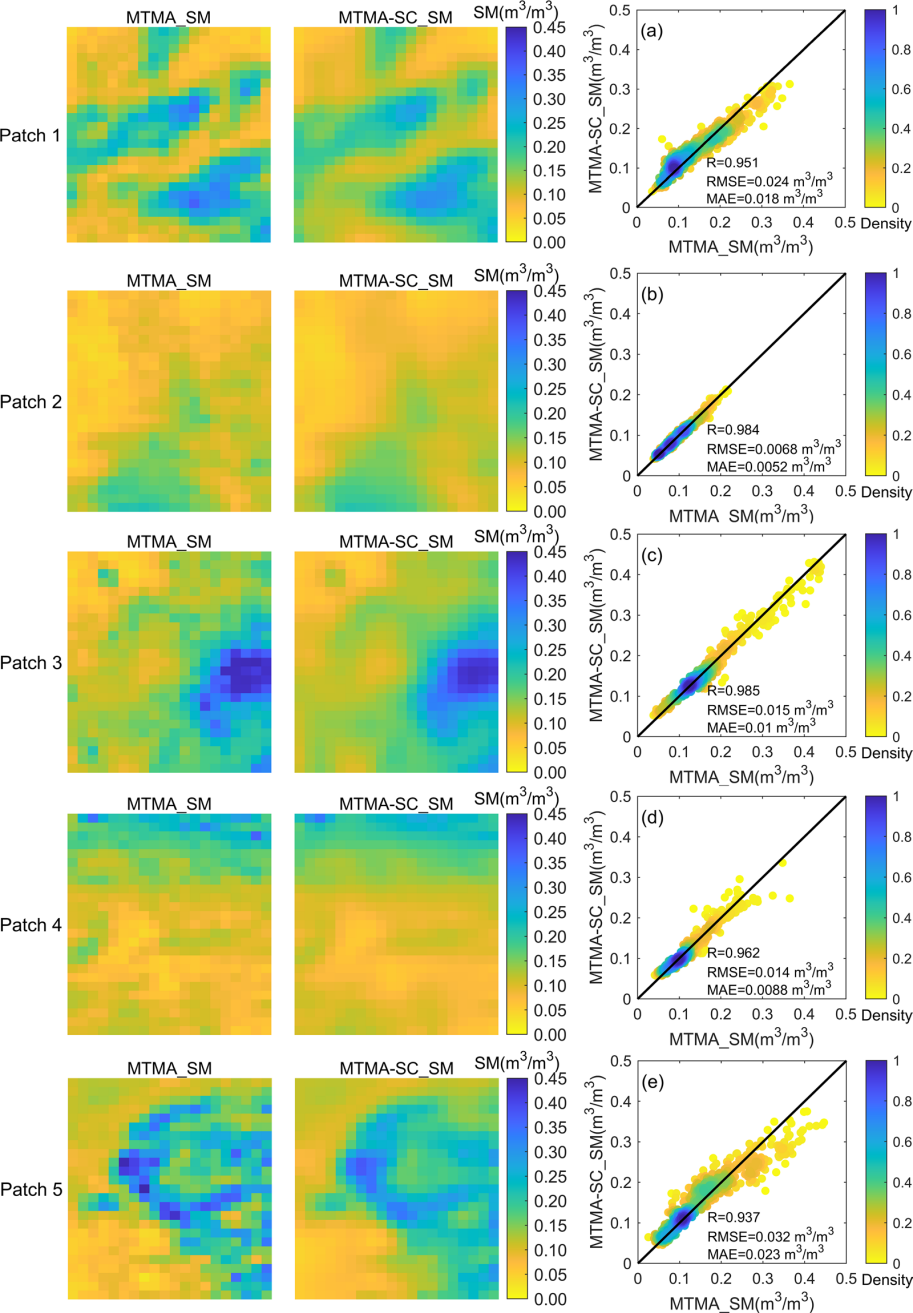
Spatial distribution and scatters of original soil moisture and reconstructed soil moisture of five simulated missing data areas in 1 June 2017: the first column represents the spatial distribution of original soil moisture products with five simulated missing data areas, the second column represents the spatial distribution of reconstructed soil moisture, and the third column represents the scatters of original soil moisture and reconstructed soil moisture of five simulated missing data areas.

In Fig. 5a,b, the reconstructed soil moisture exhibits continuous spatial texture within the five circular simulated missing data regions, respectively. Furthermore, the reconstructed soil moisture within these five circular areas also showed spatial continuity with the surrounding original soil moisture. To further illustrate the spatial distribution of the reconstructed soil moisture in the simulated missing data areas, the five selected simulated missing data regions was enlarged in Fig. 6, and compared the values in these areas with the corresponding original soil moisture. According to the first and second columns of Fig. 6, it becomes evident that the spatial distribution of the reconstructed soil moisture is closely consistent with that of the original soil moisture. This result indicates that the reconstructed soil moisture effectively reproduces the continuous characteristics of soil moisture spatial changes within the simulated missing data regions.

This study used R, RMSE, and MAE metrics to quantitatively evaluate the accuracy of the reconstructed soil moisture in simulated missing data areas. In Fig. 6a–e, it is evident that the scatter distribution of the reconstructed MTMA-SC_SM of the selected five simulated missing data regions and the corresponding original MTMA_SM closely aligns with the 1:1 line. Moreover, the correlation coefficients between the two all exceed 0.93, providing substantial evidence of the favorable consistency between the MTMA-SC_SM and the MTMA_SM. Furthermore, the RMSE and MAE of the MTMA-SC_SM of the five simulated missing data areas range from 0.0068 to 0.032 m^3^/m^3^ and 0.0052 to 0.023 m^3^/m^3^, respectively, indicating minimal deviations from the MTMA_SM, which meets the expected accuracy (0.04 m^3^/m^3^) of the SMOS satellite. Overall, the MTMA-SC_SM not only showed a high degree of agreement with the spatial distribution characteristics of the MTMA_SM in the simulated missing data areas, but also showed negligible deviations, confirming the ability of the DCT-PLS method to reconstruct spatial continuity information.

### Assessment of SMOS MTMA-SC_SM products

In the past section, this study confirmed the reliability of the DCT-PLS method. We then applied the DCT-PLS method to the SMOS MTMA_SM product, producing SMOS MTMA-SC_SM products spanning almost ten years. This section focuses on evaluating the spatio-temporal performance of the reconstructed results of the gaps in the MTMA_SM product.

#### The spatial patterns of the reconstructed product

**Fig. 7:**
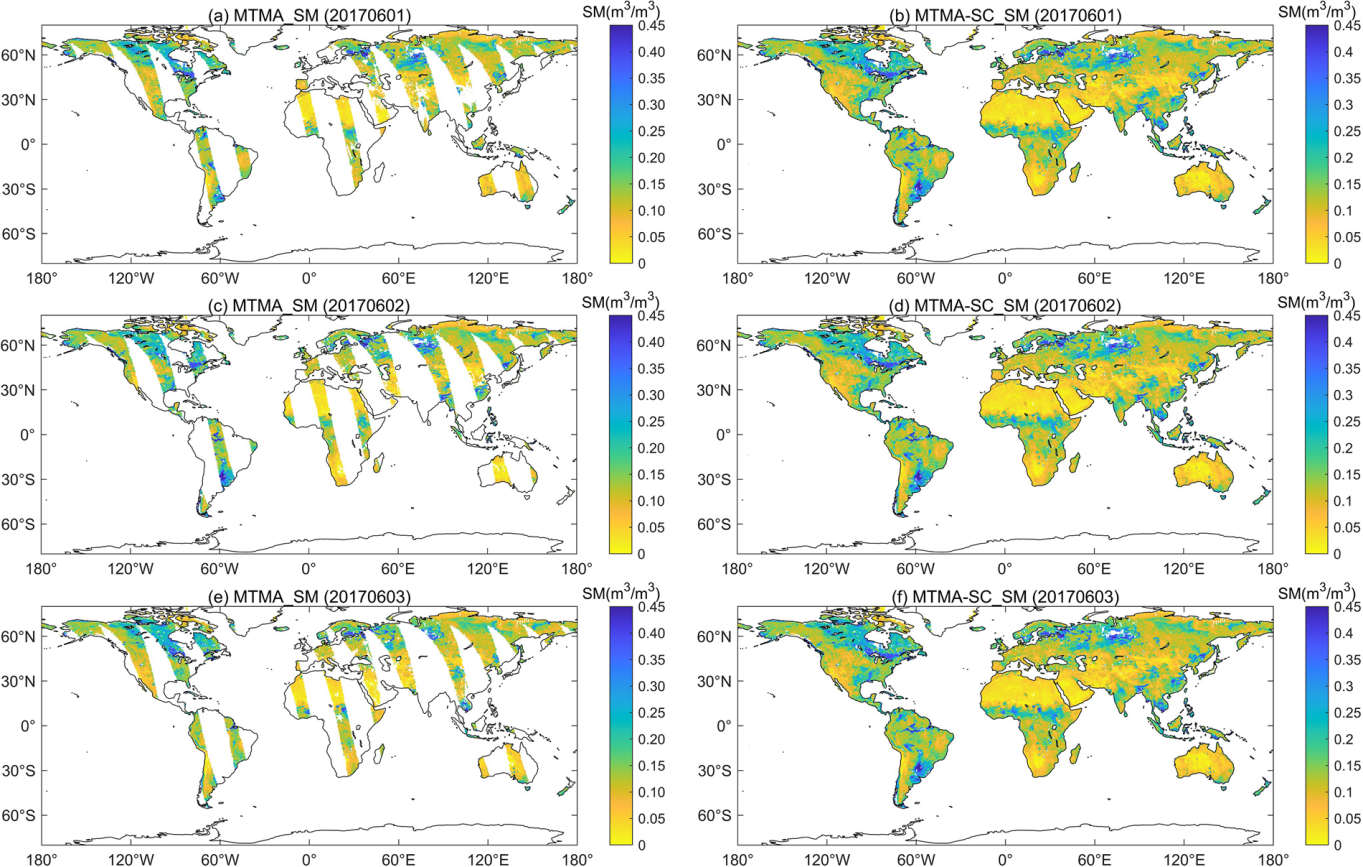
Global spatial distribution of original soil moisture and reconstructed soil moisture from 1 to 3 June, 2017 (the first column is the original soil moisture product, and the second column is the reconstructed soil moisture product).

Figure 7 displayed the global spatial distribution of the MTMA_SM and MTMA-SC_SM products from 1 to 3 June 2017. It should be noted that the MTMA method did not produce soil moisture data for certain landcover types such as water bodies, snow and ice, permanent wetlands and urban and built-up lands. Consequently, the soil moisture for these landcover types were not reconstructed using the DCT-PLS method in this study.

From the spatial perspective, the reconstructed regions of the MTMA-SC_SM product exhibit continuous spatial information with their adjacent original-valued regions (Fig. 7b,d,f). Particularly, there are no apparent boundary effects around the high- and low-valued regions, such as in the Sahel region of Africa, Australia and southern South America. Moreover, the spatial distribution of the MTMA-SC_SM product generally reflects the spatial patterns of soil moisture in different climatic regions.

From the temporal perspective, the MTMA-SC_SM product utilises the sequential time-series soil moisture information. The time-series daily MTMA-SC_SM product results (Fig. 7b,d,f) are highly similar and correlative, but there are also some variations and differences between each other.

Therefore, this study concludes that the MTMA-SC_SM product not only exhibits continuity of spatial information, implying that the spatial information within the reconstructed data region and between it and adjacent original-valued region is continuous, but also maintains the overall consistency of temporal information on spatial distribution characteristics between daily-scale soil moisture products.

**Fig. 8:**
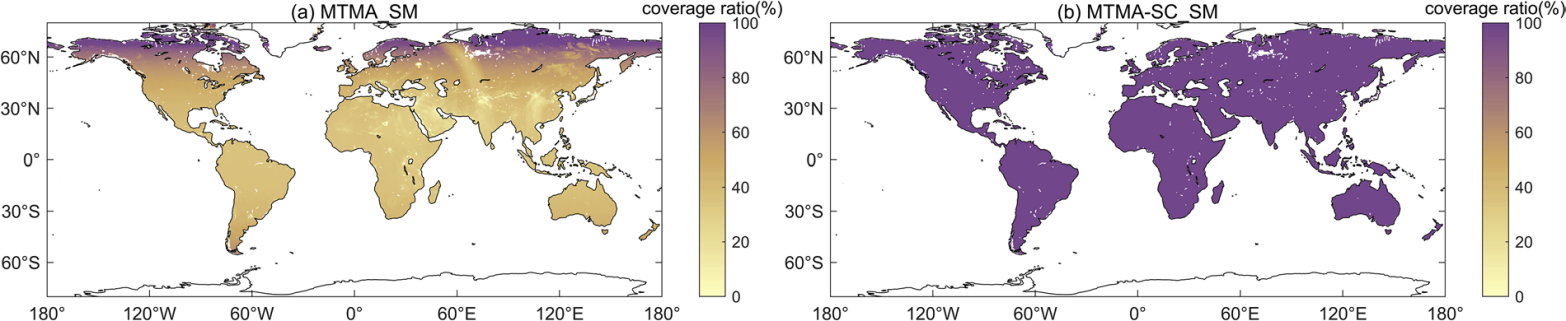
Spatial distribution of coverage rate of soil moisture retrievals in each pixel from 2015 to 2017: (**a**) MTMA_SM product and (**b**) MTMA-SC_SM product.

Figure 8 presents the global spatial distribution of coverage rate of soil moisture retrievals for each pixel on the global land surface for MTMA_SM and MTMA-SC_SM products from 2015 to 2017. It is evident that the coverage rate of MTMA-SC_SM soil moisture product (Fig. 8b) has increased to 100% compared to MTMA_SM product (Fig. 8a), demonstrating the effectiveness of the DCT-PLS method in improving spatiotemporal coverage.

**Fig. 9:**
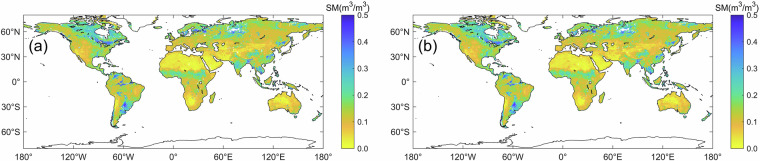
The spatial distribution of global average soil moisture of original soil moisture (**a**) and reconstructed soil moisture (**b**) from 2015 to 2017.

**Fig. 10:**
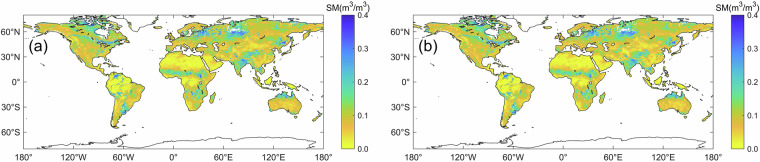
Global spatial distribution of seasonal amplitude of original soil moisture (**a**) and reconstructed soil moisture (**b**) from 2015 to 2017.

The global average soil moisture and the seasonal amplitude (2015–2017) of the original MTMA_SM and reconstructed MTMA-SC_SM products are shown in Fig. 9 and Fig. 10, respectively. The spatial distribution of the MTMA_SM and MTMA-SC_SM products exhibits similarity and generally follows the distribution pattern of the climatic regions (Fig. 9). Seasonal amplitude (SA) is defined as the difference between the values at 95% and 5% percentiles of the data after applying a 45-day moving-window-average^13,59^. Both the MTMA_SM and MTMA-SC_SM products also show agreement in the spatial distribution of seasonal amplitude in Fig. 10a,b, with relatively high amplitude values over the Indian Peninsula, the northwest of Europe, the Sahel region, which are affected by the monsoon precipitation, and relatively low amplitude values in the tropical forests of Central Africa and the Amazon areas. The similarity in seasonal amplitude between the MTMA_SM and MTMA-SC_SM products indicates that the reconstruction results do not distort the seasonal characteristics of soil moisture, while reasonably ensuring that the spatial and temporal characteristics of soil moisture changes. The above results further confirm the satisfactory performance of the MTMA-SC_SM product, with no significant deviation in the global distribution pattern compared to the original MTMA_SM product.

#### In-situ validation

This study also quantitatively evaluated the MTMA_SM, MTMA-SC_SM, and MTMA-SC_SM-Recon (a subset of the MTMA-SC_SM product containing only the gap-filled values) products using *in-situ* measurements from soil moisture networks. MTMA-SC_SM is a seamless-continuity product consisting of MTMA_SM and MTMA-SC_SM-Recon. The MTMA-SC_SM-Recon product includes both intra- and inter-swath gap-filled values, with the latter being the dominant component, as illustrated by the predominance of inter-swath gaps in Fig. 7, whereas intra-swath gaps are not prominent. We present the statistical metrics of the MTMA_SM, MTMA-SC_SM-Recon, and MTMA-SC_SM, respectively.

**Fig. 11:**
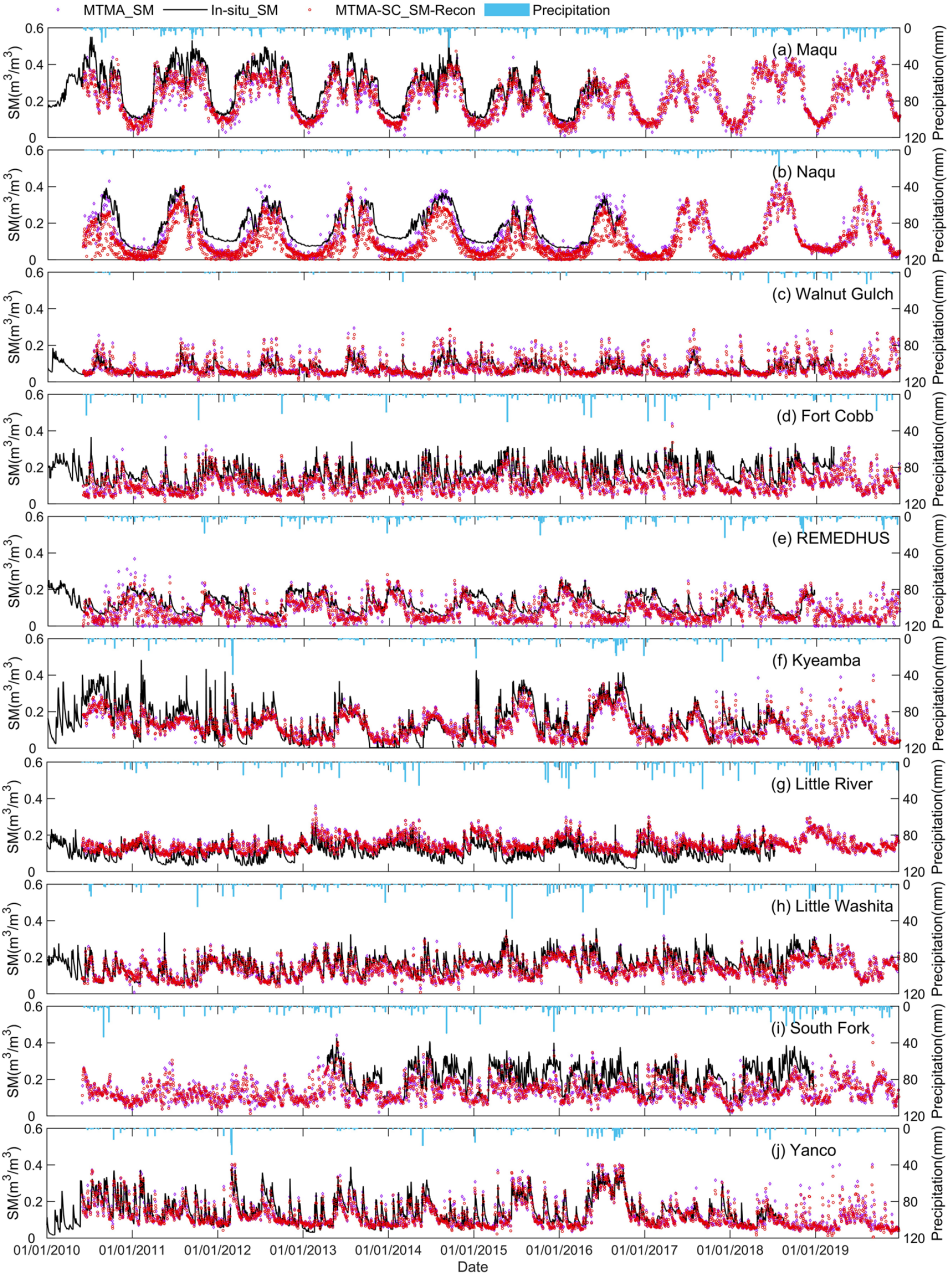
Comparison between the time series of MTMA_SM and MTMA-SC_SM-Recon in 2010 to 2019 at different ground validation networks.

The time series MTMA-SC_SM-Recon and MTMA_SM products at 10 soil moisture networks (Naqu, Maqu, Yanco, Kyeamba, Little River, Little Washita, South Fork, Fort Cobb, REMEDHUS, and Walnut Gulch) from 1 January 2010 to 31 December 2019 are shown in Fig. 11. The MTMA-SC_SM-Recon results exhibit a characteristic of continuous changes in soil moisture over time at most validation networks. Furthermore, there is also the back-and-forth consistency between the values of the MTMA_SM and the values of the temporally adjacent MTMA-SC_SM-Recon, i.e., if the values of the MTMA_SM product are high or low, its temporally adjacent values of the MTMA-SC_SM-Recon will also be high or low, such as in the Naqu and Maqu soil moisture networks. However, the MTMA-SC_SM-Recon product may not capture changes in soil moisture related to rainfall and agricultural irrigation in a timely manner, such as in the Kyeamba (Fig. 11f), South Fork (Fig. 11i) and Yanco (Fig. 11j) soil moisture networks. Despite this, the time series validation for the MTMA-SC_SM-Recon results also demonstrate the applicability of the DCT-PLS method used in this study.

The accuracy metrics of the MTMA_SM, the MTMA-SC_SM-Recon and the MTMA-SC_SM products based on *in-situ* soil moisture data from 22 validation networks are calculated. With the exception of the MySMNet and Ali networks, the R values of the MTMA_SM, the MTMA-SC_SM-Recon and the MTMA-SC_SM products exceed 0.55 at all validation networks (Supplementary Table S1), indicating that the MTMA_SM and MTMA-SC_SM products are in good agreement with the *in-situ* data. Notably, the MTMA-SC_SM product demonstrates the highest R at the Maqu and BNZ-LTER networks, exceeding 0.9 (Supplementary Table S1). Conversely, the MTMA-SC_SM product has the lowest R value in the MySMNet soil moisture network, which can be attributed to the influence of the dense vegetation (the vegetation type in the MySMNet network is evergreen broadleaf forest). For dense vegetation, the uncertainty of the MTMA_SM product obtained by the MTMA method has been discussed by Bai^17^, and then this uncertainty is also inevitably transferred to the MTMA-SC_SM product. The ubRMSE of the MTMA_SM, MTMA-SC_SM-Recon and MTMA-SC_SM products range from 0.022 to 0.057 m^3^/m^3^, 0.0025 to 0.061 m^3^/m^3^, and 0.024 to 0.058 m^3^/m^3^ (Supplementary Table S1), respectively, indicating close proximity. In addition, the overall accuracy metrics of combining data from all the networks have also been calculated for the MTMA_SM, MTMA-SC_SM-Recon and MTMA-SC_SM products. The comprehensive evaluation yielded the following overall metrics for MTMA_SM, MTMA-SC_SM-Recon and MTMA-SC_SM: R (0.733, 0.742, 0.746), ubRMSE (0.057, 0.059, 0.057) m^3^/m^3^, Bias (-0.039, -0.034, -0.035) m^3^/m^3^, and RMSE (0.069, 0.068, 0.067) m^3^/m^3^ (Supplementary Table S1), suggesting minimal differences in accuracy between the three, with a remarkably high level of consistency. On the whole, the MTMA-SC_SM product agrees well with *in-situ* soil moisture and maintains a level of accuracy comparable to the MTMA_SM product. These validation results confirm the reliability and availability of the MTMA-SC_SM product.

## Usage Notes

This study begins by introducing the principle of the DCT-PLS method and subsequently assesses its reliability for soil moisture reconstruction. Finally, the DCT-PLS method is implemented on the MTMA_SM product, and the resulting reconstructions are subjected to validation. The reliability of the DCT-PLS method for soil moisture reconstruction is demonstrated by the simulation experiments involving time series and spatial region reconstruction with missing data. And through spatiotemporal validation for the MTMA-SC_SM product, it was found that: the MTMA-SC_SM product had a good consistency with the *in-situ* soil moisture data (overall R > 0.7 and overall ubRMSE = 0.057 m^3^/m^3^), and the difference of each accuracy indicator between the MTMA_SM product and the MTMA-SC_SM product was less than 0.01; the spatial distribution of soil moisture in the gap-filled data area demonstrated continuity with the adjacent original-valued areas, without significant boundary effects.

In addition, there is room for improvement in the reconstructed soil moisture obtained by the DCT-PLS method. Soil moisture data reconstructed using the DCT-PLS method did not respond adequately to unexpected events (such as rainfall or irrigation events), and how to consider these effects in the DCT-PLS method should be further improved in subsequent research.

## Supplementary information

Supplementary Table

## Data Availability

The SMOS MTMA-SC global daily soil moisture dataset is stored in National Tibetan Plateau Data Center (http://data.tpdc.ac.cn/). The dataset is available at https://data.tpdc.ac.cn/en/disallow/821266fd-4f7f-4aa6-9d1e-79f67823f5b2.

## Code availability

The input data processing, dataset generation and validation were conducted using Matlab software (R2024b version). Code is available at https://github.com/RSBai/SMOS-SC.git after this work is accepted.
