# Supplementary material for: A decade-long seamless-continuity daily L-band soil moisture product derived from SMOS observations since 2010
Source: Sci Data. 2026 Feb 11;13:425. doi: 10.1038/s41597-026-06756-9 (PMC13004983; doi:10.1038/s41597-026-06756-9)
Supplement: Supplementary file 1 — Supplementary Table [file 41597_2026_6756_MOESM1_ESM.pdf]

Supplementary Table S1 The validation metrics of MTMA_SM, MTMA-SC_SM-Recon and MTMA-SC_SM based on in-situ data from 2010 to 2019

Network/Sites	Product	R	ubRMSE(m^3/m^3)	Bias(m^3/m^3)	RMSE(m^3/m^3)	Number
REMEDHUS	*MTMA_SM*	0.717	0.042	-0.035	0.055	1457
	MTMA-SC_SM-Recon	0.716	0.039	-0.037	0.054	1863
	MTMA-SC_SM	0.727	0.039	-0.036	0.054	3107
Yanco	*MTMA_SM*	0.797	0.048	-0.011	0.049	1361
	MTMA-SC_SM-Recon	0.839	0.042	-0.014	0.045	1846
	MTMA-SC_SM	0.824	0.044	-0.014	0.046	2942
Kyemba	*MTMA_SM*	0.795	0.056	-0.019	0.060	1282
	MTMA-SC_SM-Recon	0.830	0.053	-0.020	0.057	1748
	MTMA-SC_SM	0.819	0.054	-0.020	0.058	2853
South Fork	*MTMA_SM*	0.601	0.057	-0.070	0.090	901
	MTMA-SC_SM-Recon	0.575	0.057	-0.071	0.091	1065
	MTMA-SC_SM	0.594	0.056	-0.071	0.090	1909
Fort Cobb	*MTMA_SM*	0.832	0.032	-0.047	0.057	1402
	MTMA-SC_SM-Recon	0.747	0.038	-0.048	0.061	1976
	MTMA-SC_SM	0.785	0.036	-0.048	0.060	3217
Little River	*MTMA_SM*	0.855	0.022	0.042	0.048	1299
	MTMA-SC_SM-Recon	0.781	0.027	0.042	0.050	1955
	MTMA-SC_SM	0.814	0.025	0.042	0.049	2970
Little Washita	*MTMA_SM*	0.849	0.030	-0.021	0.037	1421
	MTMA-SC_SM-Recon	0.796	0.035	-0.021	0.041	2026
	MTMA-SC_SM	0.822	0.032	-0.021	0.039	3219
Walnut Gulch	*MTMA_SM*	0.753	0.031	0.007	0.032	1297
	MTMA-SC_SM-Recon	0.729	0.031	0.007	0.032	1975
	MTMA-SC_SM	0.740	0.031	0.007	0.032	3176
Maqu	*MTMA_SM*	0.892	0.052	-0.062	0.081	960
	MTMA-SC_SM-Recon	0.914	0.047	-0.061	0.077	1489
	MTMA-SC_SM	0.916	0.046	-0.062	0.077	2209
Naqu	*MTMA_SM*	0.884	0.050	-0.048	0.069	916
	MTMA-SC_SM-Recon	0.846	0.050	-0.086	0.100	2158
	MTMA-SC_SM	0.912	0.039	-0.070	0.080	2242
Ali	*MTMA_SM*	0.456	0.028	-0.051	0.058	870
	MTMA-SC_SM-Recon	0.494	0.025	-0.049	0.055	1456
	MTMA-SC_SM	0.483	0.025	-0.049	0.055	2152
Benin	*MTMA_SM*	0.850	0.040	0.041	0.057	545
	MTMA-SC_SM-Recon	0.829	0.042	0.039	0.058	1107
	MTMA-SC_SM	0.842	0.041	0.039	0.057	1598
Niger	*MTMA_SM*	0.771	0.045	0.026	0.052	569
	MTMA-SC_SM-Recon	0.653	0.046	0.026	0.053	1128
	MTMA-SC_SM	0.699	0.045	0.026	0.052	1653

(Continued)

Network/Sites	Product	R	ubRMSE(m^3/m^3)	Bias(m^3/m^3)	RMSE(m^3/m^3)	Number
BNZ-LTER	*MTMA_SM*	0.903	0.026	-0.077	0.081	560
	MTMA-SC_SM-Recon	0.908	0.026	-0.077	0.081	203
	MTMA-SC_SM	0.922	0.024	-0.077	0.080	771
FMI	*MTMA_SM*	0.551	0.036	-0.072	0.081	2310
	MTMA-SC_SM-Recon	0.553	0.036	-0.070	0.079	349
	MTMA-SC_SM	0.549	0.036	-0.072	0.080	2388
HOBE	*MTMA_SM*	0.594	0.037	-0.082	0.090	2004
	MTMA-SC_SM-Recon	0.563	0.039	-0.085	0.093	1530
	MTMA-SC_SM	0.590	0.038	-0.084	0.092	3208
MySMNet	*MTMA_SM*	0.478	0.052	-0.046	0.069	195
	MTMA-SC_SM-Recon	0.481	0.050	-0.047	0.069	395
	MTMA-SC_SM	0.500	0.049	-0.047	0.068	579
RISMA	*MTMA_SM*	0.671	0.056	-0.082	0.100	748
(Manitoba)	*MTMA-SC_SM-Recon*	0.620	0.061	-0.082	0.102	674
	MTMA-SC_SM	0.649	0.058	-0.082	0.100	1314
RISMA	*MTMA_SM*	0.817	0.039	0.000	0.039	747
(Saskatchewan)	*MTMA-SC_SM-Recon*	0.794	0.041	-0.003	0.041	624
	MTMA-SC_SM	0.811	0.039	-0.001	0.039	1299
SMN-SDR	*MTMA_SM*	0.633	0.043	-0.051	0.066	251
	MTMA-SC_SM-Recon	0.681	0.040	-0.054	0.067	309
	MTMA-SC_SM	0.663	0.041	-0.053	0.067	477
TERENO	*MTMA_SM*	0.641	0.056	-0.082	0.100	1459
	MTMA-SC_SM-Recon	0.651	0.058	-0.084	0.100	1471
	MTMA-SC_SM	0.648	0.057	-0.085	0.102	2769
Reynolds Creek	*MTMA_SM*	0.617	0.054	-0.045	0.071	1370
	MTMA-SC_SM-Recon	0.610	0.054	-0.047	0.072	1612
	MTMA-SC_SM	0.619	0.054	-0.046	0.071	2776
All networks	*MTMA_SM*	0.733	0.057	-0.039	0.069	23924
	MTMA-SC_SM-Recon	0.742	0.059	-0.034	0.068	28954
	MTMA-SC_SM	0.746	0.057	-0.035	0.067	48828
